# Student safety in middle school: Implications for school nurse and teacher collaboration

**DOI:** 10.1111/phn.13112

**Published:** 2022-06-25

**Authors:** Mitzi C. Pestaner, Deborah E. Tyndall, Shannon B. Powell

**Affiliations:** ^1^ Assistant Professor East Carolina University College of Nursing Greenville North Carolina

**Keywords:** adolescent health, mental health, school health, suicide

## Abstract

**Objective:**

Adolescent suicide is a public health crisis. School connectedness, a protective factor, may be especially important in low‐income rural schools, with fewer resources and higher rates of suicide as compared to urban schools. The purpose of this study was to explore teacher perceptions of safety and school connectedness in a low‐income, rural middle school, and implications for collaborative practice between school nurses and teachers.

**Design and Sample:**

A qualitative secondary data analysis was used. Data were taken from transcripts from four focus groups comprised of middle school teachers (n = 20).

**Measurement:**

An inductive approach to content analysis was conducted using in vivo Coding and Venn diagrams.

**Results:**

Three themes were identified: (1) defiant and aggressive student behaviors were safety concerns, as teachers perceived they may be obscuring mental health needs; (2) teachers were sometimes placed in a position to assist students with safety management strategies; and (3) managing safety concerns was an obstacle to building connectedness, as reflected in safety and school connectedness.

**Conclusion:**

Collaborative strategies between school nurses and teachers are essential to identify student behaviors that may be masking mental health needs. Strategies have the potential to enhance school connectedness and support student safety.

## 1 INTRODUCTION

Suicide rates among adolescents in the United States have increased by more than 50% in the past decade, creating a public health crisis (Jameson, 2020). For youth aged 10–19 years, suicide is the second leading cause of death (Centers for Disease Control & Prevention [CDC], 2019). School connectedness, the perception by students that adults and peers within the school care about them and their learning (CDC, 2009), is a significant protective factor against suicidal thoughts and behaviors in adolescents (CDC, 2018; Kim et al., 2019). School connectedness may act as a protective mechanism for at‐risk adolescents by increasing coping attitudes and behaviors, fostering the perception that adults are supportive, and increasing the likelihood that they will seek help from adults within the school (CDC, 2009; Whitlock et al., 2014). Connectedness between staff and students can be developed by cultivating an environment comprised of trusting, respectful, supportive, and caring relationships (CDC, 2009; Marraccini & Brier, 2017; Whitlock, 2006; Whitlock et al., 2014).

School connectedness may be especially important in low‐income, rural schools where there are fewer resources for students and the rate of adolescent suicide is two times higher in comparison to urban schools (Marraccini et al., 2021). School poverty, defined as schools in which the average family income is approximately $29,000, may be a significant risk factor for suicide attempts (Fang, 2018; Hirsch & Cukrowicz, 2014). As such, lower levels of school connectedness in impoverished schools may place students with mental health needs at increased risk for suicide attempts (Fang, 2018). Additionally, in schools where poverty exists, studies have found an association between lower levels of school connectedness and disruptive behavior (O'Brennan et al., 2014; Voight et al., 2015). Disruptive behaviors, such as anger or aggression, and social withdrawal, have been associated with depression (Lindsey et al., 2017). This raises the concern for safety as depression is a significant risk factor for suicide (Carballo et al., 2019).

School nurses have a significant role in collaborating with others to promote mental health and enhance protective factors (National Association of School Nurses [NASN], 2018). School nurses often collaborate with social workers, school counselors, and other clinicians to identify and refer students with suicidal risk (Allison et al., 2014; Biddle et al., 2014; Hooven et al., 2012). K‐12 teachers are an additional resource within the school setting that are well‐positioned to collaborate with school nurses to promote student safety. Because teachers have frequent and direct contact with students, they are uniquely positioned to provide surveillance of behaviors suggestive of suicide risk or “red flags” (Dimitropoulos et al., 2021; Lindsey et al., 2017), making them critical collaborators. Whereas K‐12 teachers can offer their constant observation and noticing of shifts in student behavior, school nurses possess expertise in screening and referrals to mitigate suicidal risk (NASN, 2018). While the literature is limited regarding school nurse and teacher collaboration for student mental health needs (Hoskote et al., 2022), these two professionals often collaborate on the management of chronic diseases and other medical needs of students (Anderson et al., 2018; Best et al., 2018; Schaffer et al., 2016). Since, these collaborations have had a positive impact on student health outcomes (Blackwell et al., 2017; Pufpaff et al., 2015), it is reasonable to examine how collaborative efforts might improve student outcomes related to mental health.

Previous research was conducted in a low‐income, public middle school to examine teacher perceptions of students’ emotional health needs and the use of school connectedness strategies to address these needs (manuscript under review. It was apparent during primary analysis that safety was an emerging theme that affected school connectedness. As a result, a more focused secondary analysis was warranted to examine the theme of safety and consider implications for school nursing practice. While more research is needed to learn more about school nurse and teacher collaboration in caring for student mental health needs, we first need to understand teacher concerns regarding those needs. Therefore, the purpose of this secondary data analysis was to explore teacher perceptions of safety and school connectedness in a low‐income, rural middle school and explore implications for collaborative practice between teachers and school nurses.

## 2 METHODS

### 2.1 Design

A qualitative secondary analysis (Heaton, 2008) was undertaken to investigate additional questions not explored in the primary research. One type of secondary data analysis, *supplementary analysis*, provides a more in‐depth examination of an emergent issue not fully explored in a primary study (Heaton, 2008). In this *supplementary analysis*, a more in‐depth examination of the concept of safety related to school connectedness is explored.

### 2.2 Setting

The primary study was conducted in August through November of 2019 in a rural, public middle school in the Southeast United States receiving Title 1 funding. The study site served a student body (*n *= 430) of 6th, 7th, and 8th grade students comprised of 56% African Americans, 22% Hispanics, 17% European Americans, and 5% two or more races (National Center for Education Statistics [NCES], 2019). Seventy‐two percent of the students were eligible for free or reduced‐price lunches, as compared to the state average of 44.3% (North Carolina Department of Public Instruction [NCDPI], 2018). The school's bullying and in‐school suspension rates were nine and seven times higher than the average county or state rates, respectively (NCDPI, 2019).

### 2.3 Sample

Teachers were recruited by email to participate in one of four focus groups. Twenty out of 22 (91%) teachers agreed to participate. Teachers were grouped by grade level representing a diverse sample of 6th, 7th, and 8th grades and elective classes. The majority of participants had been employed at the school 3 years or less (85%) and were female (65%), White (75%), and 45 years of age or older (55%).

### 2.4 Data collection

The primary study was approved by the university's Institutional Review Board (IRB). Participants first completed an online survey designed to capture student behaviors that could be indicative of unmet emotional health needs (e.g., bullying, defiance, aggression) and the frequency of these behaviors. Next, focus groups were conducted using semi‐structured questions to elicit further discussion of the most frequently reported behaviors. Sample questions included: *Disruptive or acting out behaviors have been reported as a common concern observed in students, what specific behaviors have you observed? What strategies do you use to address these behaviors?* Four 60‐minute focus groups were conducted and audio‐recorded at the middle school during teacher planning periods.

One mode of secondary data analysis involves the re‐use of self‐collected data (Heaton, 2008). The researchers conducting this secondary data analysis also conducted the primary study, strengthening the credibility and trustworthiness of the analysis (Ruggiano & Perry, 2019). After consultation with the IRB, it was deemed that this secondary analysis was using an existing database and did not warrant additional review. As such, we were not required to reapproach participants for consent.

### 2.5 Analytic strategy

Using clean, uncoded transcripts from the original study, data were re‐analyzed with a new perspective, the exploration of the concept of safety related to school connectedness. An inductive approach to content analysis was undertaken as the exploration of safety within the context of school connectedness was a novel way of analyzing the data (Elo & Kyngäs, 2008). Two members of the research team analyzed the data initially with in vivo Coding to construct a coding scheme (Saldaña, 2013). A third researcher joined during the second cycle coding phase in which focused coding was used to continue analysis. Abstraction involved synthesizing large sections of the data and developing broader categories and sub‐categories (Elo & Kyngäs, 2008; Giles et al., 2016; Saldaña, 2013). Using Saldaña (2013, p. 344–345) as a guide to continue thematic analysis, each researcher developed a trinity configuration to identify major themes. Overlapping circles of a Venn diagram were used to represent how each researcher visualized the relationships between themes. These visualizations facilitated dialogue to create three final themes. This iterative process involved a rigorous synthesizing and collapsing of categories into several major themes and ongoing discussion until consensus of the interpretation of the data was reached (Elo & Kyngäs, 2008).

## 3 RESULTS

The concept of safety related to school connectedness was explored to consider implications for school nursing practice. Secondary analysis of focus group data revealed three major themes: 1) *safety concerns, 2) safety management, and 3) safety and school connectedness*.

### 3.1 Safety concerns

Participants’ descriptions of student behaviors posing potential safety issues were captured in the theme, *safety concerns*. Participants expressed concern for students who may be suffering from depression and anxiety, and the potential for self‐harm. One participant reported they were aware of at least three student suicide attempts within the last year and speculated there may have been more. Participants noted that most students were guarded and were likely not to confide in them if they needed help. While most were guarded, one participant described a student who offered a covert gesture during a classroom activity:

We were playing Kahoot! and [the student] gave his nickname as ‘I cut my wrists’…and this is middle school. I knew it was a cry for help.

Other cries for help came in the form of self‐injurious “cutting” behaviors. While only discussed in two of the focus groups, participants in these focus groups acknowledged they were aware of cutting behaviors in certain students.

One participant felt most of the students at this school, if contemplating self‐harm, would not present as depressed but rather defiant and aggressive. Participants agreed that most students suppressed their emotions which eventually erupted as acting‐out behaviors in the classroom. These acting‐out behaviors were sometimes aggressive, compromising the safety of the students themselves, as well as their peers and teachers. Some of the most aggressive behaviors contributing to safety concerns included “physical fights” between students; students throwing objects in the classroom when angry; and peer bullying. Due to suppressed or displaced anger, one participant thought high‐risk students may act out and do “something extreme” like “I'm mad, I hate this school, and I'm going to go home and kill myself” [participant projecting emotion].

### 3.2 Safety management

As a result of these safety concerns, teachers often implemented strategies to manage safety. For example, one participant distributed a flyer to her students about a suicide hotline as she felt a “duty” to tell them about available resources. Other participants often found themselves as the first point of contact for students in distress:

We've had some that have their emotional breakdown in the bathroom and you just kind of stand there and wait until you can get them to breathe enough to get them out the door.

Although, participants seemed willing to manage student safety when needed, there were barriers. Lack of knowledge and training to handle students at‐risk was a concern for many. Participants questioned their own strategies asking, “Did I say the wrong thing to a student today?” or “How can I better address issues that present themselves?” One participant noted, “I have my coping mechanisms for myself [but] I feel like I'm not qualified to tell them what's best for them.” Participants also described instances in which they had little or no information about a student's mental health due to confidentiality restrictions imposed by the school district. One participant shared a situation where a student engaged in self‐injurious behavior on the day of progress reports. The participant went on to express helplessness when the student returned to school because of lack of knowledge about the student's risk for injury.

I don't think that anyone wants [a suicide] on their watch. So, it's like…was it the progress report? What was it? I think that there [were] a bunch of missing pieces that we aren't a part of and it's hard to tell what those pieces are.

There was a sense of uneasiness about being responsible for student safety, but participants utilized available resources when appropriate. When participants were concerned about their students, they referred them to the school counselor. This was a helpful resource for students; however, participants felt one counselor at the school could not meet the demand. While participants within each focus group noted the school counselor as a resource for student support, the school nurse was not mentioned.

### 3.3 Safety and school connectedness

Participants’ descriptions of student safety concerns and their inadvertent positioning to manage safety created barriers that prevented them from connecting with students. Many participants noted their lack of training in mental health impeded their ability to manage safety situations effectively. One participant expressed worry over her responses, or lack of appropriate responses, to address a student with mental health needs:

That's the thing that will keep me up; like did I say the wrong thing to a student today? Did I do anything wrong? What did I do and how did I do it wrong? How can I better address issues that present themselves?

Participants elaborated on the importance of understanding student mental health needs; however, policies regarding confidentiality were a barrier. One participant shared how not having information on students’ mental health issues and needs limited teachers’ ability to make better judgments about classroom strategies:

A kid might, just from something we perceive as simple, get up, storm out, have a meltdown; just not knowing and having a relationship with the child, not knowing more about their triggers.

Many participants expressed the belief that identifying and managing safety concerns was critical for connecting to students and thus, students’ well‐being and academic success. Connecting to students on a deeper level, beyond academics, was important to the participants. For example, one participant stated, “If you can reach a child emotionally everything else comes through. They'll be safe, they'll feel supported, they'll feel nurtured by you.” However, participants perceived a lack of training and the district's confidentiality policies were both barriers that hindered their ability to effectively manage safety risks for students. As a result, there were missed opportunities for participants to show students they cared about them and their well‐being, impacting the ability to foster school connectedness.

## 4 DISCUSSION

This study was conducted in a low‐income, rural middle school to explore teacher perceptions of student safety and the impact on school connectedness. Similar to Dimitropoulos et al. (2021), we found that teachers are often the first ones to see safety concerns that may warrant further intervention. Findings indicate that teachers are often on the frontline of safety issues and tasked with managing the safety of individual students as well as classroom safety. While often tasked with these responsibilities, barriers such as lack of training and confidentiality policies thwarted participants’ ability to effectively manage these concerns. This inability to effectively manage safety concerns hindered opportunities for participants to build trusting and caring relationships with students which are critical to school connectedness.

Participants described defiant and aggressive student behaviors which raised safety concerns. While these behaviors could be obscuring mental health needs, (Durbeej et al., 2019; Lindsey et al., 2017); defiant and aggressive behaviors are often misinterpreted as conduct issues, evoking negative reactions from teachers (Klinck et al., 2020; Lindsey et al., 2017). As a result, students may be being reprimanded with short‐term suspension for their behaviors (Quin, 2017). Notably, African American and Hispanic boys are more likely to experience suspension for disruptive and aggressive behaviors (Lindsey et al., 2017; Voight et al., 2015). This is concerning as African American middle school students have reported lower levels of connectedness and adult‐student relationships (Voight et al., 2015). In middle schools where the student body is comprised of a majority of youth of color, understanding underlying issues for disruptive behaviors is especially important for strengthening school connectedness.

Participants felt policies governing confidentiality of student mental health weakened their ability to manage safety concerns. One of the most frequently noted obstacles in school interprofessional collaboration is confidentiality surrounding mental health (Ekornes, 2015). While administrators may be aware, teachers are often not advised about a student's hospitalization for mental health treatment (Marraccini et al., 2021). Additionally, parents may not disclose to school staff that a student was hospitalized due to mental health issues out of fear their child will be labeled or excluded by peers and adults (Ekornes, 2015; Villatoro et al., 2018). If not disclosed, it is more difficult for the school to provide resources to support the student's recovery (Marraccini et al., 2021).

### 4.1 Implications

While participants reported initiating referrals to the school counselor, the school nurse was not utilized. School nurses have the skills to engage in early identification, intervention, and referral for mental health concerns (McCabe et al., 2022; NASN, 2021) and may be the only clinical resource in schools (Hoskote et al., 2022; McCabe et al., 2022). Collaboration can be beneficial as teachers spend a significant amount of time with students and are in an ideal position to observe and identify students’ behavioral and mental health changes (Ekornes, 2015; Shelemy et al., 2019). Sharing information regarding their observations of behavioral changes with the school nurse can facilitate early identification and referral of students who may have safety risks.

Referring students with mental health needs to the school nurse is crucial, particularly in rural or under‐served areas where there may be a dearth of mental health providers (Kaskoun & McCabe, 2021; Klinck et al., 2020). Referrals for at‐risk students could be facilitated by including the school nurse in team meetings in which students with patterns of disciplinary problems are discussed. This would support the creation of a collaborative plan of action to develop relationships with adolescents struggling in school, and referral of at‐risk students for services who would otherwise be missed. School nurses can also assist with the creation of student reintegration plans for those students re‐entering school following inpatient care for mental health. The plan should outline supports and interventions, management of stressors in school, and a safety plan (Marraccini et al., 2021). School nurses in collaboration with teachers could counsel parents prior to the student's discharge to ensure parents provide consent for communication between healthcare providers and the school (Finch et al., 2015). Following discharge, school nurses and teachers might regularly meet to evaluate the student's reintegration progress. These strategies facilitate teachers having sufficient knowledge about students’ mental health to support management of safety concerns (Ekornes, 2015; Hart & O'Reilly, 2018).

Lack of mental health training was noted by participants as a barrier in managing safety concerns. School nurse coordination of mental health training and education for teachers would support teachers’ abilities to identify and manage student safety concerns (Onnela et al., 2014). Additionally, collaborative conferences between the teacher, parent and school nurse to discuss concerns would promote open communication about strategies to support student safety (Kaskoun & McCabe, 2021). These collaborative conferences have the potential to enhance awareness of safety concerns and positively influence school connectedness (Madjar et al., 2018; Onnela et al., 2014; O'Shea et al., 2021). While our findings have implications for school nursing, not all school nurses will be equipped for this type of collaboration. Inadequate training, lack of administrative support, and high student caseloads may impede opportunities for collaboration. Notably, the school nurse assigned to the middle school in this study provides coverage to four other schools within the district, which may explain why participants did not mention their school nurse as a resource during focus groups.

Since mental health conditions among youth have increased during the pandemic (O'Shea et al., 2021), research that examines the impact of interprofessional collaboration within schools is critical. Research is needed to examine outcomes of teacher‐school nurse collaborative interventions aimed to enhance school connectedness and promote student safety. For example, referrals prior to and after the implementation of mental health training for teachers, and the outcome of those referrals, could be tracked. Research examining teacher‐school nurse interventions and outcomes would provide empirical evidence of the implications of collaborative interventions in promoting student safety.

### 4.2 Limitations

The findings should be considered in light of several limitations. The use of secondary analysis of data from one middle school with a relatively small sample limits the transferability of findings. We were also limited to the data collected in the primary study; therefore, participants were not specifically asked about student safety and its impact on school connectedness. Nonetheless, the secondary analysis offered new insights which generated implications for school nursing practice. Additionally, data used were collected in 2019 and does not reflect the experiences of teachers in the classroom following the COVID‐19 pandemic. Further research is needed to investigate how teacher‐school nurse collaboration might mitigate student safety concerns occurring from the impact of the pandemic.

## 5 CONCLUSION

This study offers insight into teacher and school nurse collaboration to promote student safety. Teachers are well‐positioned to provide surveillance and identify student behaviors warranting additional intervention. School nurses possess the expertise to provide additional intervention through screening and referrals. Together, these complementary roles have the potential to mitigate suicidal risk in students who might be contemplating self‐harm. These types of support structures, where students feel adults are responsive, offer support, and genuinely care about their well‐being, strengthens school connectedness. Strengthening the protective factor of school connectedness is crucial given the recent rise in adolescent suicide rates. Fostering school connectedness is especially important as the COVID‐19 pandemic continues to impact the mental health of our youth, especially in low‐income schools and communities who have been disproportionately affected by the pandemic.

## Data Availability

The data that support the findings of this study are available from the corresponding author upon reasonable request.
